# A Review of Open Access, No-Fee Journals Indexed in PubMed and Scopus for Publishing Image Reports in Medical Research

**DOI:** 10.15388/Amed.2024.31.2.21

**Published:** 2024-12-04

**Authors:** Gabriele Gaggero

**Affiliations:** Pathology Unit, IRCCS Istituto Giannina Gaslini, Genova, Italy

**Keywords:** Fees and Charges, Open Access Publishing, PubMed, Scopus, Case Reports, Raktažodžiai: Mokesčiai ir rinkliavos, atviros prieigos leidyba, „PubMed“, „Scopus“, atvejų aprašymai

## Abstract

**Background:**

Aspects of medicine can be conveyed through paradigmatic images (anatomical-surgical, radiological, microscopic) in image reports, a type of article more immediate than case reports. The aim of this review is to find journals allowing the publication of image reports and that are free of charge, open access and indexed in the best databases.

**Methods:**

The search started with a Boolean string and followed the PRISMA 2020 guidelines. Eligibility criteria were: English language, human medicine field, international DOI and ISSN codes applied, free of charge, open access, PubMed/Scopus indexed.

**Results:**

15,504,000 items were retrieved; 102 met all criteria. Instructions to authors were screened to extract journals that allowed image reports: 39 (39/39 Scopus-indexed; 29/39 PubMed-indexed). Most were in oncology (6/39) and general medicine (5/39), followed by neuroscience, fetal/pediatric and nephrology (4/39 each), urology, dermatology, hematology (3/39 each), thoracic/pleural/peritoneal diseases (2/39), and finally endocrinology, cytology, rheumatology, ophthalmology, gastroenterology (1/39 each). 21/39 allowed a single image; word count ranged from 100 to 1500. 32/39 reported a maximum number of references (range: 0–20), while 15/39 reported a maximum number of authors (range: 2–6).

**Conclusions:**

Compared to the vast publishing landscape, there are very few English-language, open access, PubMed/Scopus-indexed medical journals that allow free of charge publication of image reports. The majority are in the fields of oncology and general medicine, but other specialties are also represented. Image reports are usually articles with a limited number of words, references and authors allowed, as their purpose is much more a practical/didactic take-home message rather than a broad research with many authors. The review shows that image reports, still important for their educational value in medical knowledge transfer, are freely publishable and consultable in journals with international visibility.

## Abstract (it)

**Įvadas:**

Medicinos aspektai gali būti perteikiami paradigminiais vaizdais (anatominiais-chirurginiais, radiologiniais, mikroskopiniais) vaizdų ataskaitose, kurios yra artimesnio pobūdžio nei atvejų ataskaitos. Šios apžvalgos tikslas – rasti nemokamų žurnalų, kuriuose galima skelbti vaizdų ataskaitas, kurie yra atviros prieigos ir indeksuojami geriausiose duomenų bazėse.

**Metodai:**

Paieška pradėta nuo loginės eilutės ir vykdyta pagal PRISMA 2020 gaires. Tinkamumo kriterijai buvo šie: anglų kalba, žmogaus medicinos sritis, taikomi tarptautiniai DOI ir ISSN kodai, nemokama, atvira prieiga, PubMed/Scopus indeksas.

**Rezultatai:**

Buvo rasta 15 504 000 elementų; 102 atitiko visus kriterijus. Instrukcijos autoriams buvo patikrintos, kad būtų galima išrinkti žurnalus, kuriuose leidžiama pateikti vaizdų ataskaitas: 39 (39/39 „Scopus“ indeksuoti; 29/39 „PubMed“ indeksuoti). Daugiausia jų buvo onkologijos (6/39) ir bendrosios medicinos (5/39), toliau neurologija, vaisiaus / pediatrija ir nefrologija (po 4/39), urologija, dermatologija, hematologija (po 3/39), krūtinės ląstos / blužnies / pilvaplėvės ligos (2/39) ir galiausiai endokrinologija, citologija, reumatologija, oftalmologija, gastroenterologija (po 1/39). 21/39 leido pateikti vieną paveikslėlį; žodžių skaičius svyravo nuo 100 iki 1500. 32/39 nurodė didžiausią literatūros šaltinių skaičių (intervalas: 0-20), o 15/39 nurodė didžiausią autorių skaičių (intervalas: 2-6).

**Išvados:**

Palyginti su plačia leidybos erdve, yra labai nedaug atviros prieigos, PubMed/Scopus indeksuojamų medicinos žurnalų anglų kalba, kuriuose galima nemokamai skelbti vaizdų ataskaitas. Dauguma jų yra onkologijos ir bendrosios medicinos sričių, tačiau yra ir kitų specialybių žurnalų. Vaizdų ataskaitos paprastai yra straipsniai, kuriuose leidžiamas ribotas žodžių, nuorodų ir autorių skaičius, nes jų paskirtis yra labiau praktinė/didaktinė išvada, o ne platus tyrimas su daugybe autorių. Apžvalga rodo, kad vaizdų pranešimai, vis dar svarbūs dėl savo mokomosios vertės perduodant medicinos žinias, gali būti laisvai publikuojami ir konsultuojami tarptautinio žinomumo žurnaluose.

## Introduction

Despite the significant shift in biological research towards the ‘omics’ sciences (genomics, proteomics, transcriptomics, etc.), a substantial part of medical knowledge is still conveyed through the description of individual cases. These often aim to convey a didactic message through paradigmatic cases or to highlight clinical, surgical, radiological, pathological or genetic anomalies.

The definition and composition of case reports (CRs) have been extensively discussed [1, 2], including their scientific value [3-7], ethical considerations regarding patient consent [8], and the identification of reputable journals for publication [9-14].

An equally effective method of communicating case-related knowledge is through concise, explanatory images, known as image reports (IRs). Although IR lacks a formal definition, it can be described as a didactic or scientific message presented visually rather than narratively, prompting questions such as “What diagnosis would you make from this image?” and “What medical experiences does this evoke?” These questions stimulate curiosity and encourage readers to engage with the content of the article, which is typically short and direct.

However, it is increasingly difficult for authors to find journals that publish both CRs and IRs free of charge, especially those that are Open Access (OA) and indexed in major databases. This study aims to identify international, English-language medical journals that allow IR publication, are fully OA, have no associated fees, and are indexed in PubMed and/or Scopus.

## Methodology

The search strategy followed the PRISMA 2020 guidelines [15]. The criteria were applied across different search engines and databases, as detailed in the following lists.

1. The internet search engines/databases used were:
  - Google,
  - Google Scholar,
  - Medline,
  - Cochrane Library,
  - PubMed,
  - Scopus.
2. The search Boolean string applied to the databases was: (“medical articles” OR “medical journals” OR “medical papers” OR “medical manuscripts” OR “scientific articles” OR “scientific journals” OR “scientific papers” OR “scientific manuscripts”) AND (“without fees” OR “no fees” OR “without article processing charge” OR “without APC”).

In addition to the search criteria, the following inclusion criteria were then applied: international journal, in English, covering topics in human medicine, open access and with both Digital Object Identifier (DOI) and International Standard Serial Number Identification (ISSN) codes.

## Results

A total of 15,504,000 items were found. After removing all repetitions of identical results, all non-English language journals, all non-OA journals, and all non-human medicine journals (e.g., chemistry, physics, mathematics, computer science, plant biology, veterinary science, etc.), 152 journals were found. However, 20 of them were excluded because (1) they did not explicitly state that they were completely free of charge for online access and/or for publishing articles (so-called hybrid journals); (2) on closer examination, they actually charged a fee for community and/or subscription and/or for the publication of color images. Another criterion was applied, i.e. that the journals found were indexed in PubMed and/or Scopus (since these are the most popular databases for reasons of academic visibility), and thus another 30 were excluded, yielding 102 results. For each of these 102 journals, the TOA was examined to see if the IR was available.

**Figure 1:**
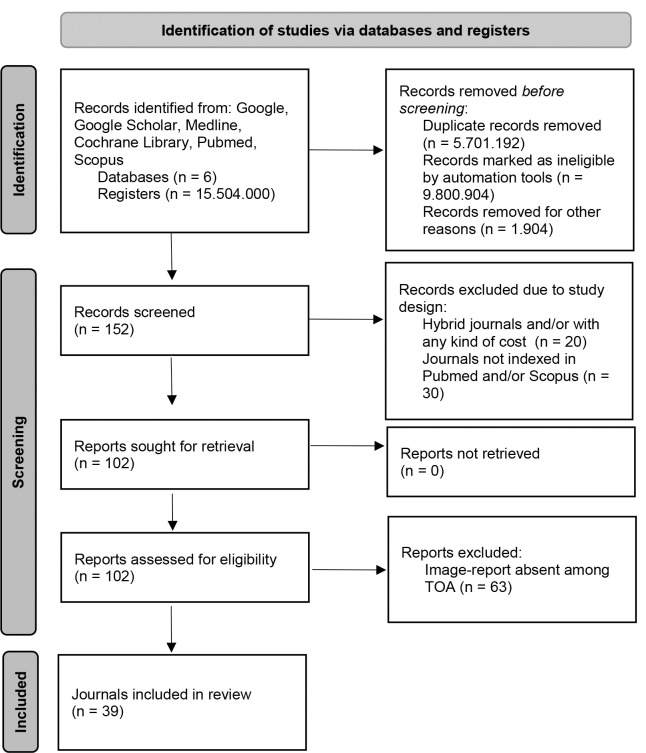
**PRISMA 2020 flow diagram for new systematic reviews which included searches of databases and registers only**. The PRISMA 2020 method was used to obtain the final result of 39 journals that met all the established criteria. Abbreviations: TOA= Type Of Article.

The final result was 39 OA scientific medical journals, English language, without fees and/or APC, and with IR among the TOA (Figure 1).

Of these, all (39/39) were Scopus-indexed and 29/39 were also PubMed-indexed. The medical specialties were: 6/39 (15.4%) oncology; 5/39 (12.8%) general medicine; 4/39 (10.3%) neuroscience; 4/39 (10.3%) fetal/pediatric; 4/39 (10.3%) nephrology; 3/39 (7.7%) urology; 3/39 (7.7%) dermatology; 3/39 (7.7%) hematology; 2/39 (5.1%) chest or pleural/peritoneal diseases; 1/39 (2.6%) endocrinology; 1/39 (2.6%) cytology; 1/39 (2.6%) rheumatology; 1/39 (2.6%) ophthalmology; 1/39 (2.6%) gastroenterology. Of these, 28/39 (71.8%) were indexed (by JCR), about half (21/39; 53.8%) allowed publication of a single image or a panel of 4 images, with a range (including all 39 journals) of 1 to 6 images. The mean number of words allowed was 528.4 and the median was 500 (with a range between 100 and 1500 words). 32/39 (82.1%) reported the maximum number of references (range between 0 and 20), while 15/39 (38.5%) reported the maximum number of authors allowed (range 2–6) (Table 1, 2 and 3).

**Table 1:** The table shows, in alphabetical order, the 39 journals obtained from the review that included image reports among their article types. The tabulated data were extrapolated by reading the instructions for authors of each selected journal. Specific instructions regarding the journal described are given in parentheses. Instead, when a data was not specified, it was tabulated here as N.I. (Not Indicated); JCR: Journal Citation Reports.

Journal name	I.F. (JCR)	PubMed indexed	Scopus indexed	Image(s) allowed by the instructions for Authors	Words count	Number of references max	Number of Authors max
Acta Haematologica Polonica	none	NO	YES	1 (1 panel with 6 pics) additional figures in supplementary data	750	10	N.I.
Acta Medica Portuguesa	1.1	YES	YES	1 (composed of 2 images)	150	6	3
African Journal of Thoracic and Critical Care Medicine	none	YES	YES	1	150	N.I.	N.I.
Annals of Indian Academy of Neurology	1.7	YES	YES	4	600	5	N.I.
Asian Journal of Urology	2.6	YES	YES	2	500	4	N.I.
Autopsy and Case Reports	none	YES	YES	4 (at least one macroscopic image required)	500	6	N.I.
Brazilian Journal of Nephrology	1.2	YES	YES	1 (1 panel with 4 pics)	100	5	3
Cancer Research, Statistics and Treatment	none	NO	YES	1	1500	10	N.I.
Clinical and Experimental Pediatrics	4.2	YES	YES	2	800	10	N.I.
Current Journal of Neurology	0.7	YES	YES	1	400	N.I.	N.I.
Current Urology	1.6	YES	YES	3	300	5	N.I.
Dementia & Neuropsychologia	none	YES	YES	2 (and 1 table)	750	20	N.I.
Dermatology Practical and Conceptual	2.8	YES	YES	1	250	2	6
Endocrinology and Metabolism	3.4	YES	YES	1	1 page A4 format	5	N.I.
Fetal and Pediatric Pathology	1.1	YES	YES	1	‘text no longer than a half page‘	5	N.I.
Gazi Medical Journal	0.1	NO	YES	1	250	none	N.I.
Hematology, Transfusion and Cell Therapy	2.1	YES	YES	N.I.	100	3	3
Indian Journal of Cancer	1.0	NO	YES	1	400	3	3
Indian Journal of Medical Research	4.2	YES	YES	1	150	none	2
Indian Journal of Nephrology	0.8	YES	YES	2	500	5	N.I.
Indian Journal of Urology	1.1	YES	YES	2 (2 images or tables)	500	5	3
Journal of Applied Hematology	none	NO	YES	1	200	2	3
Journal of Cutaneous and Aesthetic Surgery	none	YES	YES	1	800	6	N.I.
Journal of Cytology	1.3	YES	YES	1	800	6	N.I.
Journal of Rheumatic Disease	2.0	YES	YES	1 (1 panel with 4 pics)	500	5	N.I.
Journal of Neurocritical Care	none	NO	YES	2 (template provided by the journal)	200	4	4
Kidney Research and Clinical Practice	3.0	YES	YES	2	400	none	N.I.
New England Journal of Medicine	158.5	YES	YES	1 (also more images in a single panel)	150	none	2
Nowotwory. Journal of Oncology	none	NO	YES	2	300	2	4
Oncology in Clinical Practice	0.5	NO	YES	1 (1 panel with 4 pics)	700	5	5
Oman Journal of Ophthalmology	none	YES	YES	4-5 images	1000	5	N.I.
Pleura and Peritoneum	1.8	YES	YES	N.I. (number of figures as required)	1200	5	2
Portuguese Journal of Gastroenterology	0.9	YES	YES	4	500	5	6
Reports of Practical Oncology and Radiotherapy	1.2	YES	YES	1 (1 panel with a maximum of 4 pics)	750	5	5
Surgical and Cosmetic Dermatology	none	NO	YES	6	1200	5	N.I.
Turkish Archives of Pediatrics	1.2	YES	YES	1 (and 1 table)	400	10	N.I.
Turkish Journal of Nephrology	0.1	NO	YES	8	500	5	N.I.
Tzu Chi Medical Journal	1.5	YES	YES	4 (4 images and/or tables)	500	N.I.	N.I.
World journal of Pediatric Surgery	0.8	YES	YES	1	800	10	N.I.

**Table 2:** Distribution of the different medical specialities among the 39 identified journals that allow the publication of image reports.

Medical Specialty	Count (out of 39)	Percentage
Oncology	6	15.4%
General Medicine	5	12,8%
Neuroscience	4	10,3%
Fetal/Pediatric Medicine	4	10,3%
Nephrology	4	10,3%
Urology	3	7.7%
Dermatology	3	7.7%
Hematology	3	7.7%
Chest or Pleural/Peritoneal Diseases	2	5.1%
Endocrinology	1	2.6%
Cytology	1	2.6%
Rheumatology	1	2.6%
Ophthalmology	1	2.6%
Gastroenterology	1	2.6%

**Table 3:** Main characteristics (for publication purposes) of the 39 identified journals.

Characteristic	Value	Percentage
Total Journals	39	100%
Scopus-indexed Journals	39	100%
Journals Reporting Maximum References	32	82.1%
PubMed-indexed Journals	29	74.4%
Journals Indexed by JCR	28	71.8%
Journals Indicating Allowable Images	21	53.8%
Journals Reporting Maximum Authors	15	38.5%
Range of Images Allowed	1 to 6	-
Mean Number of Words Allowed	528.4	-
Median Number of Words Allowed	500	-
Range of Words Allowed	100 to 1500	-
Range of References	0 to 20	-
Range of Authors	2 to 6	-

## Discussion

The scientific community has faced the challenge of making medical knowledge available online to as many readers as possible. Consequently, much of the scientific publishing industry has transitioned to open access (OA) models. The Bethesda Statement on Open Access Publishing defines OA as an agreement where authors and copyright holders grant users free, irrevocable access to works, allowing for copying, distribution, and the creation of derivative works, provided proper attribution is given [16]. While this definition protects the reader’s rights, it does not account for the financial burden often placed on authors, who may be required to pay publication fees to disseminate their articles as open access.

Given the ongoing need to convey medical knowledge through the description of individual cases, the scientific community has made substantial efforts to define the characteristics that an article must meet to qualify as a case report (CR). At the 2013 International Congress on Peer Review and Biomedical Publication, the CARE (CAse REport) Statement and Checklist was introduced. This tool has since been adopted by numerous medical journals and translated into various languages. Additionally, there is a wealth of literature – including articles, websites, and blogs – highlighting the educational and scientific value of CRs, as well as guidelines on where and how to write and publish them. These resources also categorize journals as paid or free, open access, or indexed in major databases. Moreover, discussions around ethical and legal considerations, such as patient privacy and the necessity of informed consent, have gained prominence in the literature.

Conversely, no comprehensive evaluation has been conducted on these aspects within the context of image reports (IRs). This gap is noteworthy because IRs differ from classical CRs. While CRs tend to provide more detailed, structured narratives of individual cases, IRs offer concise, iconographic summaries that can be equally impactful. The findings from this review suggest that, despite the extensive landscape of medical publishing, very few English-language, open access, PubMed/Scopus-indexed journals permit the free publication of IRs.

### Specialties Represented

The analysis reveals a diverse representation of medical specialties among the 39 journals reviewed. Oncology (15.4%) and general medicine (12.8%) emerged as the most frequently represented fields. This indicates the significant role of imaging in these areas, where visual documentation is essential for effective diagnosis and treatment. Other specialties, such as neuroscience, pediatric medicine, and nephrology, also contributed to the variety, underscoring the growing interest in image-report publications across multiple domains. The representation from pediatric specialties highlights the importance of clear visual communication in cases involving children, where imagery can enhance understanding.

### Journal Characteristics

All the journals included in this review are indexed in Scopus, ensuring their credibility and accessibility. In addition, 74.4% of these journals are also indexed by PubMed and 71.8% by Journal Citation Reports (JCR), increasing their visibility and robustness within the scientific community. The high percentage of journals specifying a maximum number of references (82.1%) contrasts with the lower percentage (38.5%) that indicate the maximum number of authors allowed. This discrepancy suggests a standardized approach to referencing, which reflects the rigorous academic standards expected in medical publishing. However, the variability in author limits may influence collaborative research efforts.

The majority of journals (53.8%) indicate the number of images permissible for submission. While all journals allow image submissions, the lack of clarity regarding submission limits may hinder authors’ ability to select suitable journals for their work. Standardizing this information could streamline the submission process and improve author experience.

The range of images permitted (1 to 6), along with the mean and median word counts (528.4 and 500, respectively), indicates a balance between brevity and the need for comprehensive descriptions in image reports. The flexibility in word count (ranging from 100 to 1500) further accommodates both concise and detailed submissions.

### Limitations and Future Directions

Several limitations must be acknowledged. This study focused exclusively on journals that are completely free to publish and that are indexed in Scopus and/or PubMed. If the search had been extended to include journals indexed in other major databases, such as Web of Science (WOS), Embase and Google Scholar, or to include hybrid journals that allow free publication in return for a commitment to OA, the results could have been significantly larger. The exclusive focus on English-language journals may also exclude valuable contributions from non-English publications. Future research could benefit from widening the scope to include a more diverse range of journals and languages.

Examining trends in the publication of image-based reports over time can also provide valuable insights into the evolution of medical documentation. Understanding how journals are adapting to the increasing importance of visual communication will be essential for future developments in the field.

## Conclusion

In conclusion, the data underline the diversity of medical specialties involved in the publication of image reports and the characteristics of journals that facilitate these submissions. The findings highlight the need for clarity in submission guidelines and for journals to adapt to the growing importance of visual documentation in medical practice. By fostering an environment conducive to image-based submissions, journals can improve the quality of medical literature and contribute to improved communication within the healthcare community.

## Data Availability

The data used to support the results reported in this study was extracted from the major online search engines.
